# Alternative Splicing: Role in Cancer Development and Progression

**DOI:** 10.1155/2013/421606

**Published:** 2013-11-20

**Authors:** Claudio Sette, Michael Ladomery, Claudia Ghigna

**Affiliations:** ^1^Dipartimento di Biomedicina e Prevenzione, Università di Roma Tor Vergata, Via Montpellier 1, 00133 Roma, Italy; ^2^Fondazione Santa Lucia, Laboratorio di Neuroembriologia, Via del Fosso di Fiorano 64, 00143 Roma, Italy; ^3^Faculty of Health and Life Sciences, University of the West of England, Coldharbour Lane, Bristol BS16 1QY, UK; ^4^Istituto di Genetica Molecolare, Consiglio Nazionale delle Ricerche (IGM-CNR), Via Abbiategrasso 207, 27100 Pavia, Italy

Alternative splicing of precursor messenger RNAs (pre-mRNAs) is a fundamental step in the regulation of gene expression. This processing step of the nascent messenger amplifies the coding potential of eukaryotic genomes by allowing the production of multiple protein isoforms with distinct structural and functional properties. The advent of high-throughput sequencing techniques has recently revealed that alternative splicing of exons and introns represents a major source of proteomic diversity in complex organisms characterized by a limited number of protein-coding genes. Nevertheless, the evolutionary advantage provided by alternative splicing can also turn into a source of deleterious problems for the organism. Indeed, the extreme flexibility of its regulation, which relies on the combinatorial action of multiple non stringent factors, is subject to errors and the aberrant splicing of key genes can result in the onset of many human genetic and sporadic diseases. In this regard, mounting evidence illustrates how changes in alternative splicing patterns of specific genes is an important tool used by cancer cells to produce protein isoforms involved in all areas of cancer cell biology, including numerous aspects of tumor establishment, progression, and resistance to therapeutic treatments. Importantly, cancer-specific splice variants have the potential to become suitable therapeutic targets for human cancer, as novel tools to correct splicing defects are being developed and, in some cases, have entered clinical trials for other human diseases, such as spinal muscular atrophy. Nevertheless, these findings are likely to represent just the tip of the iceberg and important questions regarding the role of alternative splicing in cancer still remain to be addressed.

The main focus of this special issue is to emphasize key mechanisms involved in oncogenic splicing changes, their connection with other steps of gene expression, and the therapeutic potential of cancer-associated alternative splicing isoforms.

More specifically, M. Ladomery discusses alternative splicing in the context of the so-called hallmarks of cancer, originally proposed by Hanahan and Weinberg in 2000. The list of hallmarks was originally six; recently it was augmented to ten. M. Ladomery proposes that a comprehensive dysregulation of alternative splicing could, in itself, be considered yet another hallmark of cancer. The idea is that the aberrant expression and activity of key oncogenic splicing factors and/or their regulatory kinases could lead to a systematic change in gene expression by favouring the concurrent production of several oncogenic splice variants of genes involved in critical biological aspects of tumour cells.

S. C. Lenzken et al. review our current knowledge of the role of alternative splicing in the multiple and various aspects of the DNA damage response (DDR) and the control of genome stability. This review illustrates several mechanisms through which pre-mRNA splicing and genomic stability can influence each other and contribute to tumorigenesis.

M. Romano and colleagues draw attention to the function that pseudoexons and pseudointrons can play directly in cancer pathology. These sequences can be found in genes that have well-established roles in cancer, including *BRCA1, BRCA2, NF-1, *and* ATM*. They describe the mechanisms through which pseudoexons and pseudointrons can be activated or repressed. In addition, they discuss their potential use as tumour biomarkers to provide a more detailed staging and grading of cancer.

C. Naro and C. Sette discuss the key role that reversible phosphorylation plays in the regulation of alternative splicing. Both splice factors and core components of the spliceosome are affected by phosphorylation. The review focuses on the role of protein kinases and phosphatases whose activity has specifically been linked to aberrant alternative splicing associated with neoplastic transformation. Moreover, it illustrates the fact that signal transduction routes that are frequently altered in cancer cells, such as the RAS/ERK and the PI3K/AKT pathways, can modulate alternative splicing events through the direct or indirect phosphorylation of splicing regulatory proteins. Thus, protein kinases and phosphatases involved in this step of gene expression regulation may provide exciting opportunities for novel drug design.

A. Best et al. describe the emerging role of Tra2*β*, an SR-related protein, in human cancer. The gene encoding this splicing factor is amplified in various types of cancer and the increased expression of Tra2*β* is associated with cancer cell survival. Interestingly, the Tra2*β* gene is a transcriptional target of the proto-oncogene ETS-1, whereas known Tra2*β* splicing targets play key roles in cancer cells, where they affect metastasis, proliferation, and cell survival. These observations point to regulation of Tra2*β* expression in cancer cells as an important step in tumorigenesis.

The review by Z. Siegfried et al. gives a series of specific examples that cover misregulated alternative splicing events affecting both the Ras-MAPK and PI3K-mTOR signalling pathways during carcinogenesis. These pathways show extensive crosstalk and are commonly altered in many cancers by genetic and epigenetic aberrations. This article also addresses how these signalling pathways play key roles in the transmission of extracellular signals to the splicing machinery and to specific RNA-binding proteins that ultimately regulate exon definition events.

C. Jackson et al. give an overview on a topic of significant clinical interest: the roles (often opposed) of the HER2 splice isoforms in breast cancer progression and drug resistance.

M. P. Paronetto describes the function of the Ewing Sarcoma protein (EWS) in cancer biology. EWS is best known for its involvement in translocations associated with sarcomas. Recent evidence has implicated EWS in the regulation of DNA damage response (DDR) in cancer. EWS is a multifunctional protein thought to help coordinate multiple steps in the synthesis and processing of pre-mRNAs. This review illustrates in detail the biochemical features and the physiological roles of this RNA binding protein and provides some hints on its possible contribution to human cancer.

Other two reviews give a series of specific examples of cancer-associated splicing variants. C. Sette discusses the growing evidence that dysregulated alternative splicing is a major factor in the remarkable biological heterogeneity of prostate cancer. Key genes, including the androgen receptor itself, are alternatively spliced, thereby expressing isoforms with opposing functions. The review also illustrates how the regulation of alternative splicing is likely to present novel opportunities in the diagnosis, prognosis, and treatment of prostate cancer. S. Bonomi et al. deal with novel information on how alternative splice variants of many cancer-related genes can directly contribute to the oncogenic phenotype, focusing on a number of processes involved in cancer progression, such as response to hypoxia, migration, invasion, and metastasis. Furthermore, they discuss some significant examples of alternative splicing isoforms selectively expressed by tumors and not by normal tissues, which may not only represent diagnostic and prognostic tumor biomarkers, but also provide potential targets for the development of new therapeutic strategies.

In their article, L. Spraggon and L. Cartegni focus on the role of U1 snRNP, an essential component of the splicing machinery, in the regulation of alternative polyadenylation and they strongly emphasize its implications in cancer pathogenesis. Moreover, this review underlines the interesting possibility of manipulating this U1 snRNP function for anticancer therapeutic purposes.

Lastly, S. Barberan-Soler and J. M. Ragle give an overview of the advantages of using the nematode Caenorhabditis elegans to study splicing regulation *in vivo*. Importantly, a large percentage of genes undergo alternative splicing also in this simple and genetically useful organism. A big proportion of these events are functional, conserved, and under strict regulation across development, suggesting that their investigation is likely to provide general mechanisms of regulation that can be applied also to human genes. Notably, the review illustrates several examples of alternatively spliced genes that have human homologues implicated in cancer biology.

We hope that this special issue will attract the attention of researchers on new progresses in the fields of alternative splicing and cancer. In particular, the articles presented herein might highlight how this posttranscriptional mechanism of gene expression plays important roles in the generation of oncogenes and tumor suppressors, describe its interplay with signaling pathways, and suggest how our knowledge of these processes is leading to a better comprehension of malignant transformation, thus helping develop novel therapeutic strategies for the treatment of cancers.

*Claudio Sette* *Claudio Sette* *Michael Ladomery* *Michael Ladomery* *Claudia Ghigna* *Claudia Ghigna*

